# MRI-based brain tumor segmentation using FPGA-accelerated neural network

**DOI:** 10.1186/s12859-021-04347-6

**Published:** 2021-09-07

**Authors:** Siyu Xiong, Guoqing Wu, Xitian Fan, Xuan Feng, Zhongcheng Huang, Wei Cao, Xuegong Zhou, Shijin Ding, Jinhua Yu, Lingli Wang, Zhifeng Shi

**Affiliations:** 1 State Key Laboratory of ASIC an System, Fudan University, Shanghai, China; 2 School of Information Science and Technology, Fudan University, Shanghai, China; 3 Huashan Hospital Affiliated to Fudan University, Shanghai, China; 4 School of Computer Science, Fudan University, Shanghai, China

**Keywords:** Brain tumor segmatation, FPGA acceleration, Neural network

## Abstract

**Background:**

Brain tumor segmentation is a challenging problem in medical image processing and analysis. It is a very time-consuming and error-prone task. In order to reduce the burden on physicians and improve the segmentation accuracy, the computer-aided detection (CAD) systems need to be developed. Due to the powerful feature learning ability of the deep learning technology, many deep learning-based methods have been applied to the brain tumor segmentation CAD systems and achieved satisfactory accuracy. However, deep learning neural networks have high computational complexity, and the brain tumor segmentation process consumes significant time. Therefore, in order to achieve the high segmentation accuracy of brain tumors and obtain the segmentation results efficiently, it is very demanding to speed up the segmentation process of brain tumors.

**Results:**

Compared with traditional computing platforms, the proposed FPGA accelerator has greatly improved the speed and the power consumption. Based on the BraTS19 and BraTS20 dataset, our FPGA-based brain tumor segmentation accelerator is 5.21 and 44.47 times faster than the TITAN V GPU and the Xeon CPU. In addition, by comparing energy efficiency, our design can achieve 11.22 and 82.33 times energy efficiency than GPU and CPU, respectively.

**Conclusion:**

We quantize and retrain the neural network for brain tumor segmentation and merge batch normalization layers to reduce the parameter size and computational complexity. The FPGA-based brain tumor segmentation accelerator is designed to map the quantized neural network model. The accelerator can increase the segmentation speed and reduce the power consumption on the basis of ensuring high accuracy which provides a new direction for the automatic segmentation and remote diagnosis of brain tumors.

## Background

Brain glioma is the most common malignant tumor caused by the cancerization of glial cells in the brain and spinal cord. It has the characteristics of high incidence, high recurrence, high mortality and low cure rate. The purpose of brain tumor segmentation is to separate the tumor tissue from the healthy brain tissue such as white matter, gray matter and cerebrospinal fluid [1]. It plays an important role in the diagnosis and treatment of the brain glioma.

The glioma image segmentation is helpful for surgical planning and can improve the survival rate. Currently, most of the segmentation of brain tumor images is performed by physicians. In clinical practice, due to the increasing number of brain tumor images, manual segmentation of different areas of brain tumors becomes an error-prone and time-consuming task for physicians. In addition, physicians’ cognitions may have different influences on the formulation of subsequent treatment plans and operations. Therefore, automated methods are needed for high accuracy brain tumor location and segmentation.

In the past, CPU was used to complete computations in CAD systems. Due to the unsatisfactory speed of processing data by the CPU, many GPU acceleration methods have been gradually studied [2, 3]. For some machine learning algorithms in bioinformatics, FPGA acceleration has also become a new direction [4, 5].

### Related work

Automated brain tumor segmentation has attracted widespread attention in the research community and has been continuously studied. Before 2010, most researchers used standard image processing methods, such as threshold-based method [6] and region-based method [7]. Suzuki et al. used an iterative thresholding algorithm for segmentation [6], but when the image contrast is low, it becomes difficult to select the threshold. In 2005, it was proved that region growth is an effective brain tumor segmentation method. Compared with other non-region-based methods, the amount of computation is less, especially for homogeneous tissues and regions [7]. Although they are simple to implement and have small amount of computation, the segmentation accuracy does not meet the practical expectation. Hence it is mostly used for two-dimensional segmentation only [8]. Subsequently, machine learning [9] has been gradually applied to medical image analysis. Many researchers have proposed brain tumor segmentation based on classification or clustering methods [10–12]. Fletcher-Heath et al. used an unsupervised fuzzy clustering algorithm, which combines domain knowledge and image processing technology to achieve tumor segmentation [10]. Zhou et al. proposed a method based on one-class support vector machine(SVM) to extract brain tumors from Magnetic Resonance Imaging(MRI) [11]. Subbanna et al. presented a fully automated hierarchical probabilistic framework for segmenting brain tumor based on multiwindow Gabor filters and an adapted Markov Random Field (MRF) framework [12]. Compared with conventional segmentation methods, these methods can improve accuracy. However, methods with higher accuracy are still needed in clinical practice.

In the past ten years, with the huge increase in computing power, deep learning methods have continued to advance. Deep neural networks can thoroughly learn hierarchical features from input images instead of pre-defined manual features. There are many well-known deep learning networks, such as Convolutional Neural Networks(CNNs) and Recurrent Neural Networks(RNNs), which are gradually applied in various tasks of medical image analysis, such as breast image analysis [13] and chest X-ray image analysis [14]. At the same time, the segmentation of brain tumors based on deep learning networks have also aroused the interest of researchers.

In 2014, D. Zikic et al. studied the possibility of directly applying CNNs to brain tumor segmentation, which achieved higher segmentation accuracy than traditional machine learning methods [15]. In 2016, Brosch et al. proposed a segmentation method based on a deep 3D convolutional encoder network, which composes of two interrelated paths, namely a convolution path and a deconvolution path. Each image contains a repetitive structure with corresponding changes. Therefore, only a few images are needed to train a network [16]. In 2017, a multi-path CNN network for brain tumor segmentation was proposed as an extension of single-path feedforward CNN [17]. Multi-path CNN can extract different features from different modalities. In 2019 and 2020, Muhammad Sharif and Javaria Amin et al. proposed several brain tumor segmentation algorithms [18–21] to further improve the segmentation accuracy and reduce the processing time. Our design is based on the 3D U-Net network proposed in [22], which extends the U-Net network [23] and replaces all 2D operations with 3D operations. It is a multi-path CNN network which can achieve great accuracy in the segmentation task.

### Challenge

Although significant progress has been made in the brain tumor segmentation, there are still problems and challenges to be solved. Firstly, brain gliomas are mutations of glial cells. Due to the wide spatial distribution of glial cells, gliomas can appear anywhere in the brain. Moreover, the shapes and sizes of brain tumors in different patients have great uncertainty, which means before segmentation process, almost no prior information can be provided to describe the shape and size of a tumor. Location uncertainty and morphological uncertainty have brought great challenges to accurately locate brain tumors. Secondly, MRI which provides the tissue details can be imaged in multiple directions. The 3D imaging method is more conducive to the detection of brain tumors, so we mainly focus on MRI to segment brain tumors. However, the MRI computation in the automated process is complicated, which usually requires more time for image analysis. There is demanding performance requirement for the segmentation processing platform.

It can be concluded that deep learning methods can achieve high accuracy in the brain tumor segmentation task. However, the brain tumor segmentation process may consume significant time and computing resources. Therefore, speeding up the CNN-based 3D brain tumor segmentation is the key to high accuracy brain tumor detection and obtaining detection results efficiently. In order to speed up the segmentation process, GPU is currently used for brain tumor segmentation, but it can be further improved in terms of speed and power consumption.

CNN realizes brain tumor segmentation in two processes: training and inference. Training is an iterative process to train the parameters. During the training process, the output of the model is compared with the expected result to update the parameters to minimize the difference. This process is repeated until the output results converge to a value that reduces the gap to an acceptable range. Training is a typical offline operation which can be done in advance. The inference process is real-time, so our work is focused on accelerating the inference process.

### Neural network for brain tumor segmentation

**Fig. 1:**
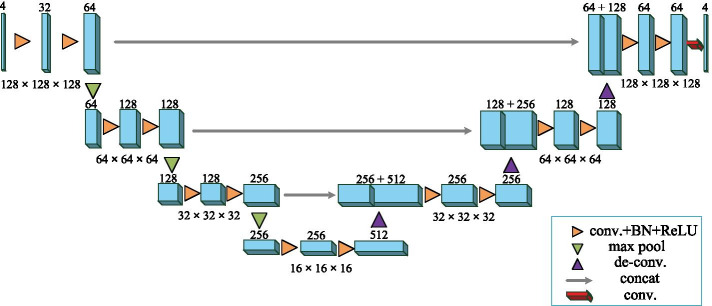
The 3D U-Net structure

Figure 1 illustrates the 3D U-Net structure which includes 3D convolutional layers, 3D deconvolutional layers, pooling layers, activation layers, and batch normalization(BN) layers. The numbers on the blue boxs are the numbers of channels, and the numbers below are the resolutions. Similar to U-Net, 3D U-Net consists of an analysis path on the left and a synthesis path on the right. The analysis path on the left includes 3D convolutional layers, BN layers and rectified linear unit (ReLU) layers. The maximum pooling layers are used to reduce the sizes of the feature maps. The synthesis path on the right includes 3D convolutional layers, 3D deconvolutional layers, BN and ReLU layers. Different from the analysis path on the left, the synthesis path expands the sizes of feature maps through the deconvolutional layers.

The 3D U-Net can provides the depth information of 3D medical images, witch has higher segmentation accuracy compared with 2D CNN segmentation networks. At the same time, it also brings large amount of computations and parameters. Therefore, it is necessary to speed up the processing of CNN-based brain tumor to realize rapid and high-accuracy brain tumor segmentation.

## Method

We have designed an FPGA-based brain tumor segmentation inference accelerator which speeds up the segmentation process. It can be shown that our FPGA-based design outperforms traditional computing technologies such as CPU and GPU implementations. In this section, we firstly describe the hardware and software platforms, and the dataset. Then the quantization process for brain tumor segmentation neural network and the hardware acceleration architecture are presented.

### Hardware and software platforms

**Fig. 2:**
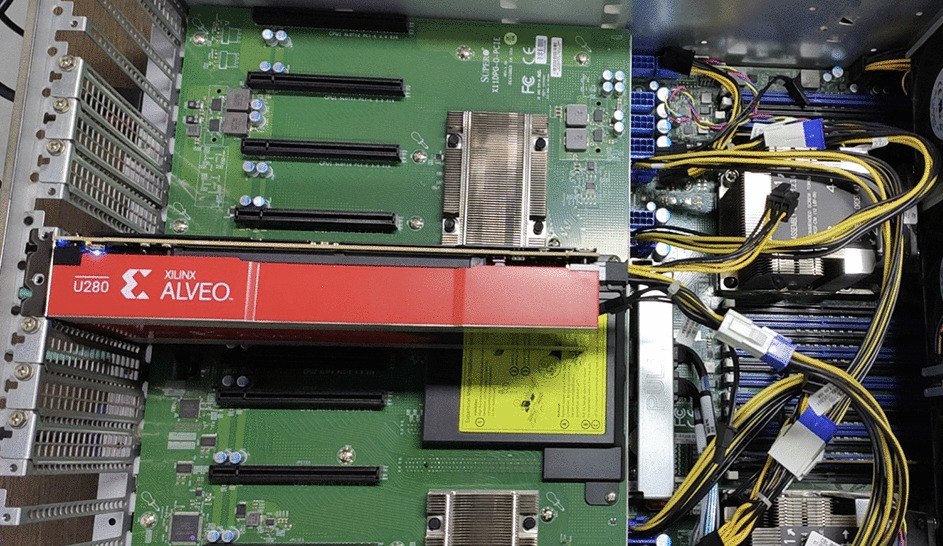
Hardware platform for brain tumor segmentation where the red is a Xilinx Alveo U280 accelerator card

*Hardware:* The neural network hardware accelerator for brain tumor segmentation is based on Xilinx’s Alveo U280 accelerator card, which has 1304k LUTs, 2607k registers and 9024 DSP slices. The hardware platform of our accelerator is shown in Fig. 2. The NVIDIA TITAN V GPU and the Intel Xeon CPU E5-2620 V4 CPU are used for comparison.

*Software* Xilinx Vivado integrated environment and Synopsys VCS simulator are used for hardware design and simulation. GPU reference designs based on single-precision floating point are compiled by CUDA 10.1 with PyTorch of python 3.8.3 and cuDNN 7.6.3 library.

### Dataset

**Fig. 3:**
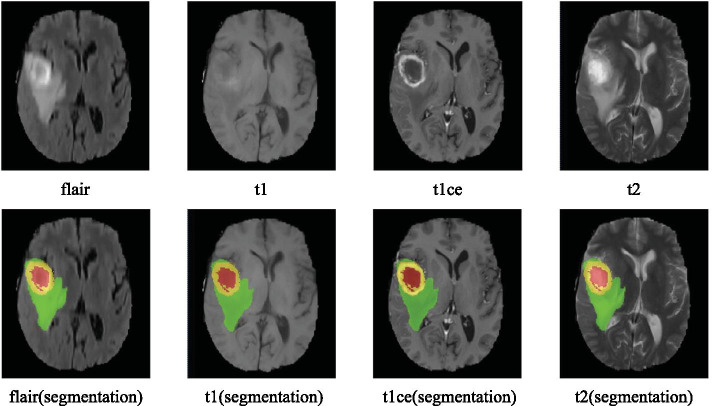
Input images with four MRI modalities and the corresponding segmentation output

The BraTS19 and BraTS20 dataset [24–26] are used to test the performance of brain tumor segmentation with four modalities for each case. A single modality of brain tumor may lead to inaccurate segmentation because it does not provide detailed information. Multi-modality images can complement each other effectively, which can improve the segmentation accuracy. Figure 3 shows one exemplar with four MRI modalities, *flair*, *t*1, *t*2 and *t*1*ce*. Each represents a unique MRI modality. The image on the right is the segmentation result in Fig. 3.

### Segmentation neural network quantization

In order to reduce the computational burden and implement the network in the FPGA hardware, it is necessary to quantize the model. The quantization process of 3D U-Net includes the following three steps [27]: merging BN layers, quantizing network parameters, and quantizing network activity data. The above three steps are detailed as follows.

#### Merging the BN layers

The BN layers in 3D U-Net can improve the training quality, but is not required for the inference. Before quantization, the BN layer can be merged into the previous convolutional layer to simplify the network structure and reduce the amount of network computations and the number of parameters. Equations 1 and 2 describe the merging process of the convolutional layer and the BN layer. In Eq. 1, *X* and *Y* represent the input and output of the convolutional layer, and *W* and *B* are the weight and bias of the convolutional kernel. In Eq. 2, $\mu$, $\sigma$, $\gamma$, $\beta$, and $\epsilon$ represent input average, input standard deviation, scale factor, offset, and a decimal to prevent the denominator from zero, respectively.

1 $$\begin{aligned} Y= & {} W \cdot X + B \end{aligned}$$

2 $$\begin{aligned} \hat{x_i}= & {} (x_i - \mu ) / \sqrt{\sigma ^{2} + \epsilon }, \nonumber \\ y_i= & {} \gamma \hat{x_i} + \beta \end{aligned}$$

By combining Eqs. 1 and 2 , the parameters after merging the BN layer can be obtained as shown in Eqs. 3, 4. Equation 5 describes the output of the merged layer.

3 $$\begin{aligned} W_{merged}= & {} W \cdot \frac{\gamma }{ \sqrt{\sigma ^{2} + \epsilon }} \end{aligned}$$

4 $$\begin{aligned} B_{merged}= & {} (B - \mu ) \cdot \frac{\gamma }{ \sqrt{\sigma ^{2} + \epsilon }} \end{aligned}$$

5 $$\begin{aligned} Y= & {} W_{merged} \cdot X + B_{merged} \end{aligned}$$

During the merging process, the 3D U-Net structure is traversed to find adjacent convolutional layers and BN layers. The merged weights and bias are obtained according to Eqs. 3 and 4 . Then the original convolutional layer and BN layer are replaced by the merged convolutional layer. The core computation equation of the deconvolutional layer is the same as that of the convolutional layer, so the above merging method is also effective for BN layers and deconvolutional layers.

#### Quantizing network parameters

After merging the BN layers, the network parameters are quantified. The network parameters adopt dynamic fixed-point linear quantization, and the quantization results are 8-bit signed fixed-point numbers. For a weight tensor *W*, such as the weight of a certain convolutional layer, we firstly determine its maximum and minimum values, then calculate scaling factors and map all the values of this tensor to the representation range of an 8-bit signed fixed-point number, that is − 128 to 127. In order to facilitate the FPGA to perform scaling operations, the scaling factors are constrained to be power of 2, so that the scaling operations can be achieved through the shift operations. The calculation of the bit number of the shift operations $b_s$ is shown in Eq. 6 where the bit width $b_w$ is 8, |*W*| is the absolute value of W, *ceil* and *max* are the round-up and the maximum functions respectively.

6 $$\begin{aligned} \begin{aligned} b_s = b_w - 1 - ceil(log_2(max(\vert W \vert ))) \end{aligned} \end{aligned}$$

In the quantization process of 3D U-Net, the weight and bias scale factors of each 3D convolutional or deconvolutional layer are calculated separately.

#### Quantizing network activity data

Network activity data are the input and output of each layer. It also uses dynamic fixed-point linear quantization, and the quantization results are signed 8-bit fixed-point numbers. A small amount of calibration data are used to run the network, and then the maximum and minimum values of the input and output of each layer are determined, finally the bit number of the shift operations of the input and output of each layer are calculated according to Eq. 6.

The process of the dynamic fixed-point linear quantization is as follows. Firstly, due to the fine granularity of the TensorFlow network model description, quantization is not convenient. The network model is rewritten by PyTorch. Secondly, the convolutional/deconvolutional layers are replaced with the corresponding merged layers. Thirdly, the network is set to training mode to use two patient data as the calibration input to run the network. This step can calculate the maximum absolute value of the input and output of each quantization layer. Finally, the quantization function of each quantization layer is run to perform quantized computation of network parameters and network activity data. The quantified network structure and network parameters are generated.

The quantization layer is implemented by the quantization decorator class INQ, which adds other operations to the forward propagation function of the quantization convolutional and deconvolutional layers. The maximum absolute value of the input and output data in the training mode is calculated. In the evaluation mode, the input and output data are dynamically fixed-point linear quantization to simulate the FPGA behavior of running the quantization network. The quantization decorator class also defines a quantized member function to calculate the bit numbers of the shift operations in the parameters and activity data, as well as the quantized parameters.

We train the 3D U-Net with the Adam optimizer and Cross-Entropy loss, the learning rate of 0.001, the batch size of 2 and the training epoch of 30. Table 1 depicts the learning hyper parameters for the segmentation model. Then the original and quantized network are tested on BraTS19 and BraTS20. The evaluation mode is set to use the test data as input to run the network forward, and calculate the pixel accuracy score (ACC) and dice similarity coefficient score (DSC). Compared with the network before the quantization, the loss is almost negligible. After quantization, the activity data and parameters are 8-bit signed fixed-point numbers, which can reduce storage resources and implement in the PFGA hardware efficiently.

**Table 1:** The learning hyper parameters for the segmentation model

Optimizer	Epoch	Batch size	Learning rate
Adam	30	2	0.01

### Accelerator architecture and design implementation

**Fig. 4:**
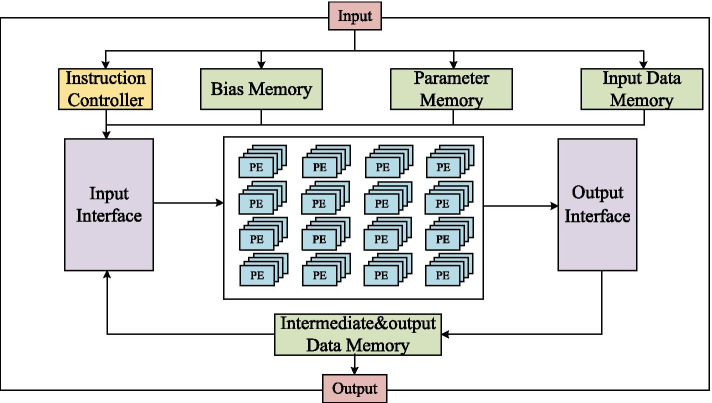
Brain tumor segmentation accelerator architecture based on FPGA

Figure 4 is an overview of the proposed accelerator. A total of 8 parts are included, which are Instruction Controller, Bias Memory, Parameter Memory, Input Data Memory, Intermediate & Output Data Memory, Input Interface, Output Interface, and Processing Element (PE) Array. After the Instruction Controller receives the configuration command, it stores the command in the configuration register, and the PE Array automatically reads data from the Bias Memory, Parameter Memory and Input Data Memory according to the configuration information, then writes the computation results in the ready storage area in Intermediate & Output Data Memory.

Due to the large difference in the number of input and output channels in each layer of the 3D U-Net, the fixed input and output channel architecture leads to the reduction of computing resources utilization. In order to solve this problem, there are three computing modes in the configurable PE architecture. These modes are 4-32, 32-32, 32-64. When the input channel of the first layer is small, we configure PE as the 4-32 mode, which computes 4 pixels on the feature map of 4 input channels and 32 output channels. For some convolutional layers and deconvolutional layers with 32 input channels and 32 output channels, we configure PE as the 32-32 mode to compute two pixels of the feature map at a time. There is also the 32-64 mode, which is used in the layer where the input channel are 32 and the output channel are 64. One pixel of the feature map is computed at a time. Three configurable modes allow our FPGA accelerator to make better use of computing resources and minimize the proportion of idle multipliers.

#### Control mechanism

**Fig. 5:**
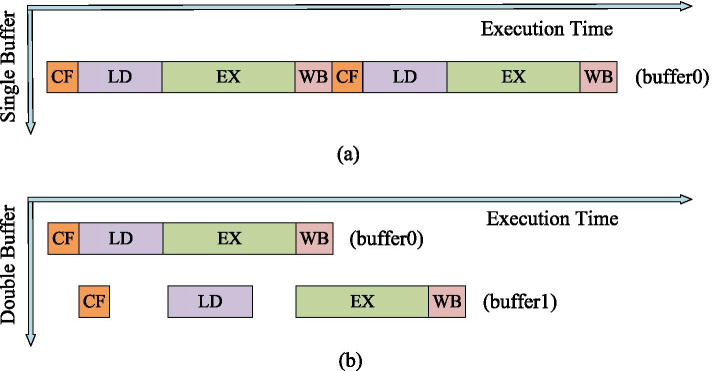
**a** The execution of each stage without the double-buffering strategy, **b** the execution of each stage under the double-buffering strategy

The execution of each instruction of the MIPS CPU is divided into 5 stages: instruction fetch (*IF*), instruction decode (*ID*), execute (*EX*), memory access (*MEM*), and register write back (*WB*). Usually these 5 stages are made into one 5-stage pipeline, and the execution of each stage of the pipeline is fixed at 1 clock cycle, so that the pipeline improves the processing speed. Our accelerator learns from the CPU’s approach and divides the execution of each instruction into 4 stages: configuration (*CF*), load data (*LD*), execution (*EX*), and write back (*WB*). Because the numbers of cycles consumed by these 4 stages are different and not fixed, it cannot form a pipeline like the MIPS CPU. The double-buffering strategy is adopt, which can make the stages with the longest cycles to cover up the time of other stages. Figure 5a, b are diagrams of the execution stages without and under the double-buffering strategy. This is the case where the execution time of the *EX* stage is longer than the other 3 stages. Under the double-buffering strategy, it can be seen that when multiple such instructions are executed, the *EX* stage will mask most of the running time of *CF*, *LD*, and *WB*, reducing the overall computation time significantly. Similarly, if *LD* becomes the longest time for each stage, the *LD* stage can cover most of the running time of *CF*, *EX* and *WB*.

#### Partition and scheduling

The current convolutional neural network accelerators can be divided into two categories. One type is multi-layer computation, that is, after completing a block of a layer, the result of this block is obtained and then the next layer is performed. The results of this layer are not written to the off-chip memory. The other is to compute layer by layer which means calculating the next layer after completing one layer. The results are not written to the off-chip memory. Since the amount of 3D U-Net parameters is tens or even hundreds of times higher than that of a 2D network. The first approach would have too many on-chip parameters. As a result, the second method of layer-by-layer computation is chosen. As we know, FPGA computing and storage resources are limited, one layer of 3D U-Net must be devided into several blocks. Different partition results will have different effects on the execution efficiency of the CNN model on FPGA. Constraints are set according to chip resources and other conditions to obtain partition result. According to the partition result, address allocation is performed on the input feature map of the first layer and the static data such as the weights and bias data of each layer.

After obtaining the partition result and the data addresses respectively, we start to schedule the execution stages. The goal of scheduling is to try to conceal the load data time or the computation time of the computation block to improve the efficiency of the execution pipeline. Firstly, we use the layer as a unit to generate a sequence of computation blocks according to the partition result. In this step, the computation process of each layer is divided into several computation blocks in a certain order. Secondly, according to the instruction set configuration rules of the chip and the label information of each computation block, we generate the instructions of the binary computation block sequence. Finally, the binary instructions are stored in the external memory for the instruction acquisition module of the design to obtain the relevant instructions.

#### Implementation and optimization of 3D convolution

**Fig. 6:**
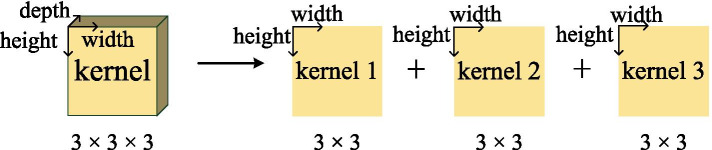
3D convolutional kernel translates into 2D convolutional kernel

In order to implement 3D convolution more conveniently, we convert 3D to 2D to speed up the computation process. For example, as shown in Fig. 6, for a $3\times 3\times 3$ convolutional kernel, the depth direction is divided into three different $3\times 3$ convolutional kernels, and the final result is obtained through the accumulation of the intermediate results of the 3 different blocks. In this way, the 3D convolution is realized through the 2D hardware architecture without affecting the computation result.

In the process of generating a sequence of computation blocks, the specific optimizations on certain layers are performed to improve efficiency. For example, due to the zero-filling operation in the deconvolutional layer, there are many zeros in the output feature map of the deconvolutional layer in 3D U-Net. In the next layer of the deconvolutional layer, the computation block whose input feature map are all zeros can be skipped to improve computation efficiency and reduce power consumption.

## Result

**Table 2:** Performance of proposed method

Dataset	Method	DSC	Execution time	Energy Consumption
BraTS19	GPU method before quantization	0.873	0.78 s	96 W
	GPU method after quantization	0.871	0.64 s	72 W
	FPGA method	0.871	0.15 s	45 W
BraTS20	GPU method before quantization	0.885	0.78 s	97 W
	GPU method after quantization	0.882	0.65 s	74 W
	FPGA method	0.882	0.15 s	45 W

**Table 3:** Proposed method comparison

Method	Year	Dice (DSC)	Average execution time
[28]	2018	0.84	2–4 min
[29]	2018	0.95	5.50 s
[30]	2019	0.85	15.25 s
[21]	2020	0.89	0.71 s
Our FPGA method	–	0.88	0.15 s

*Performance* The BraTS19 and BraTS20 datasets are used to train the network and test. In BraTS19, we use 240(HGG/LGG) cases for training and 100 cases for testing. In BraTS20, 260(HGG/LGG) cases are for training and 109 cases are for testing. The DSC, execution time, and energy consumption of our method are shown in Table 2. In BraTS19 and BraTS20, our FPGA method achieves 0.871 DSC and 0.882 DSC. The average execution time is 0.15s and the FPGA energy consumptions is 45W.

Table 3 is comparison provided with the recent method which also test on the BraTS dataset. It demonstrates that the suggested method provided accurate and efficient segmentation results. Moreover, our FPGA approach also reduces power consumption by more than half compared to CPU or GPU solutions.

**Fig. 7:**
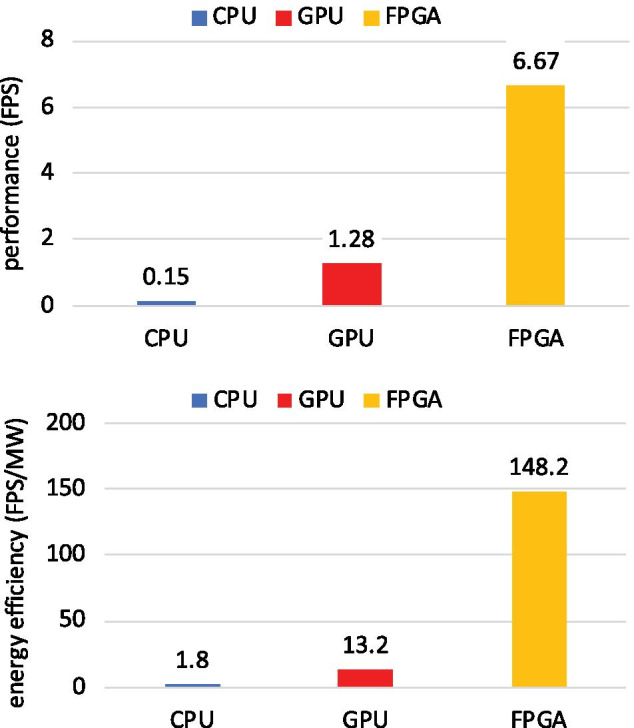
Performance and energy efficiency comparison among CPU, GPU, and FPGA

The performance and energy efficiency of FPGA, CPU and GPU designs are also compared. The execution time of testing a single image is accidental because it is too short and often not accurate enough. Therefore, the number of test images per second are measured by testing 200 MRIs segmentation tasks in BraTS19 and BraTS20 datasets to obtain the average. Performance is measured by the number of frames per second (FPS) of images processed, and energy efficiency refers to the ratio of performance and power (FPS/MW) in the computation process. Figure 7 shows the comparison of performance and energy efficiency among FPGA, CPU and GPU implementations. It can be seen that the performance of FPGA is 5.21 times higher than CPU and 44.47 times faster than GPU, and the energy efficiency ratio is 11.22 times of GPU and 82.33 times of CPU. Both processing speed and energy efficiency have been greatly improved.

**Fig. 8:**
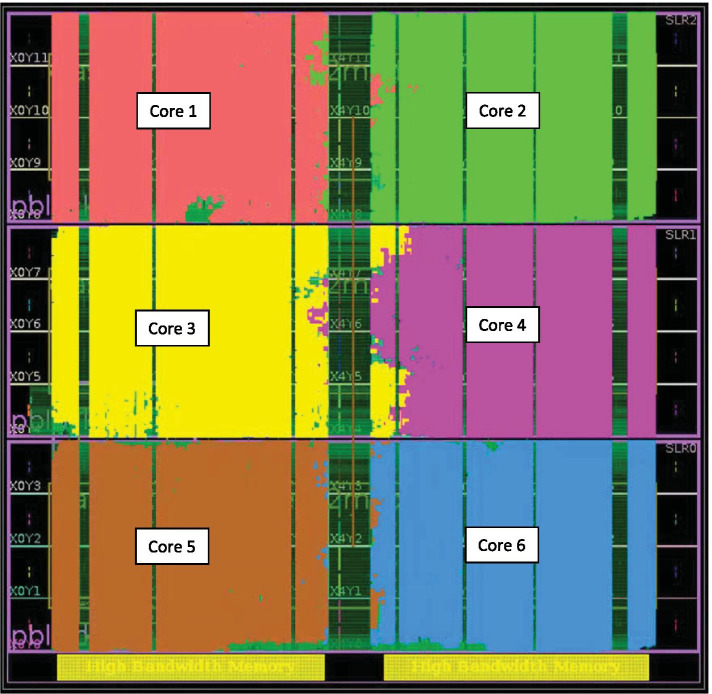
FPGA floorplan after placement and routing

*Resource usage* Our design is tested on the U280 card. The FPGA resource utilization is listed in Table 4. It can be seen that our FPGA design uses storage and computing resources reasonably. Figure 8 is the floorplan of the FPGA-based design after placement and routing. There are 6 computation cores inside the FPGA chip. Each of them is marked in a different color.

**Table 4:** Resource utilization after FPGA placement and routing

Resource	Utilization	Available	Utilization %
LUT	819956	1303680	62.90
FF	1365308	2607360	52.36
BRAM	1504	2016	74.60
URAM	648	960	67.50
DSP	5760	9024	63.83

## Discussion and future work

With the development of living environment and medical conditions, health care has become more concerned. As an important means of diagnosis and treatment by physicians, medical images have become popularized. Brain tumor segmentation as an significant part of medical image processing has also attracted the attention of researchers. The brain tumor segmentation algorithm based on deep learning has the characteristics of high accuracy and automatic learning, which breaks through the limitations of traditional brain image segmentation algorithm and becomes a hot research topic in the field of brain image segmentation in recent years. In order to improve the performance of deep neural network, researchers have made efforts on GPU platform due to the problems of large computation and complex storage in deep neural network model. GPU acceleration can accomplish the task, but the speed can still be improved. The neural network model for brain tumor segmentation can be simplified in a hardware-friendly way without affecting the accuracy of the model. As a result, FPGA design can achieve higher speed and energy efficiency than CPU and GPU.

Although brain tumor segmentation tasks are accelerated, there are still some challenges for future work. During the study, we find that the medical images of brain tumor segmentation had a relatively large sparsity, with an average sparsity of 70$\%$. If the sparse characteristic of input image can be used in algorithm or hardware implementation to save the time of invalid computation, better performance will be achieved.

## Conclusion

Brain tumor is one of the most common cancers which has the characteristics of high morbidity, high recurrence, and high mortality. Brain tumor segmentation is a very effective method to identify potentially cancerous tissue. However, this increases the burden on physicians, and the physicians’ status and experiences greatly affect the analysis results. Therefore, many CAD systems have been developed. In these systems, the first step of high-precision brain tumor segmentation is crucial for the subsequent treatment process. We propose and implement an FPGA-based brain tumor segmentation inference accelerator, which can speed up segmentation and reduce power consumption. Based on BraTS19 and BraTS20, the performance and power consumption of our FPGA accelerator are better than traditional computing technology. The average speed is 5.21 times and 44.47 times higher than that of CPU and GPU. In addition, the energy efficiency is 11.22 times and 82.33 times higher than that of CPU and GPU. The design of FPGA acceleration hardware provides a new direction for the improvement of automated brain tumor segmentation.

## Data Availability

The dataset analyzed during the current study are from BraTS, which are available at https://www.med.upenn.edu/cbica/brats-2019 and https://www.med.upenn.edu/cbica/brats2020.

## Abbreviations

CPU: Central processing unit
GPU: Graphics processing unit
FPGA: Field programmable gate array
CAD: Computer-aided detection
SVM: Support vector machine
MRI: Magnetic resonance imaging
MRF: Markov random field
CNN: Convolutional neural network
RNN: Recurrent neural network
